# Mutations in the splicing factor *SF3B1* are linked to frequent emergence of HLA-DR^low/neg^ monocytes in lower-risk myelodysplastic neoplasms

**DOI:** 10.1038/s41375-024-02249-z

**Published:** 2024-04-17

**Authors:** Susann Winter, Marie Schneider, Uta Oelschlaegel, Giulia Maggioni, Elena Riva, Marco Gabriele Raddi, Sara Bencini, Benedetta Peruzzi, Desmond Choy, Rita Antunes Dos Reis, Esther Güse, Christopher Lischer, Julio Vera, Jessica A. Timms, Nicolas Sompairac, Katja Sockel, Antonella Poloni, Antje Tunger, Matteo Giovanni Della Porta, Valeria Santini, Marc Schmitz, Uwe Platzbecker, Shahram Kordasti

**Affiliations:** 1 Department of Internal Medicine I, University Hospital Carl Gustav Carus, Faculty of Medicine Carl Gustav Carus, TU Dresden, Dresden, Germany; 2 Department of Hematology, Cellular Therapy, Hemostaseology and Infectious Disease, University of Leipzig Medical Center, Leipzig, Germany; 3 Comprehensive Cancer Centre, School of Cancer and Pharmaceutical Sciences, King’s College London, London, UK; 4 IRCCS Humanitas Research Hospital, Rozzano, Milan, Italy; 5 MDS Unit, Hematology, AOU Careggi - Department of Experimental and Clinical Medicine, University of Florence, Florence, Italy; 6 Flow Cytometry Diagnostic Center and Immunotherapy (CDCI), AOU Careggi, Florence, Italy; 7 Laboratory of Systems Tumor Immunology, Friedrich-Alexander-Universität Erlangen-Nürnberg, and Universitätsklinikum Erlangen, Erlangen, Germany; 8 Deutsches Zentrum Immuntherapie and Comprehensive Cancer Center Erlangen-EMN, Erlangen, Germany; 9 Department of Clinical and Molecular Sciences, Università Politecnica delle Marche, Ancona, Italy; 10 Institute of Immunology, Faculty of Medicine Carl Gustav Carus, TU Dresden, Dresden, Germany; 11 National Center for Tumor Diseases (NCT); German Cancer Research Center (DKFZ); Faculty of Medicine and University Hospital Carl Gustav Carus, TU Dresden; Helmholtz-Zentrum Dresden–Rossendorf (HZDR), Dresden, Germany; 12 Department of Biomedical Sciences, Humanitas University, Pieve Emanuele, Milan, Italy; 13 German Cancer Consortium (DKTK), Partner Site Dresden, and German Cancer Research Center (DKFZ), Heidelberg, Germany; 14 German MDS Study Group (D-MDS), Leipzig, Germany; 15 Haematology Department, Guy’s Hospital, London, UK

**Keywords:** Myelodysplastic syndrome, Myelodysplastic syndrome

## To the Editor:

Somatic mutations in the splicing factor *SF3B1* occur in about one-third of all myelodysplastic neoplasms (MDS) and define a subgroup of patients characterized by ring sideroblasts (RS), ineffective erythropoiesis, and an indolent disease course in lower-risk (LR) MDS [1]. They are typically heterozygous missense substitutions, most commonly (>50% in MDS) involving p.K700E (*SF3B1* NM_012433.4: c.2098A>G (p.Lys700Glu), hereafter referred to as *SF3B1*^K700E^), and have been shown to induce mis-splicing of key genes throughout erythroid differentiation [2, 3]. Surprisingly, although *SF3B1* mutations are known to target multipotent lymphomyeloid hematopoietic stem cells and clonally propagate to myeloid progenitors [4], their impact on mature immune cells remains largely unexplored. Clinically, *SF3B1* mutations are associated with high response rates to the erythroid maturation agent luspatercept and lower response to immunosuppressive treatment (IST) [5–7].

In this study, we performed multiplex immunophenotyping in conjunction with machine learning-based analytical approaches on bone marrow (BM)/peripheral blood (PB) samples from newly diagnosed or disease-modifying treatment-naïve *SF3B1*^mut^ or *SF3B1*^wt^ MDS patients (experimental cohort: Supplementary Table S1; Fig. S1) and healthy donors (HD) to identify genotype-immunophenotype correlations. Initial gene expression profiling of 730 immune-related genes in *SF3B1*^mut^ versus *SF3B1*^wt^ MDS BM mononuclear cells (BM-MNCs) revealed a predominantly myeloid cell-related innate immune gene signature (e.g., *CYBB*, *CSF1R*) lacking signs of overt myeloid-driven inflammation (i.e. *IL1B*, *CXCL5*), whereas lymphoid-related genes were underrepresented (e.g., *CD3D*, *CD79A*) (Supplementary Table S4, Fig. S2). These results are consistent with the reported lower proportion of lymphocytes in BM [8], mild myeloid dysplasia [9], and our previous finding of significantly lower *IL1B* mRNA in BM monocytes from *SF3B1*^mut^ LR-MDS [10]. As IL-1β protein levels in paired BM plasma samples were often below the detection limit, we could not determine whether lower mRNA levels correspond to lower cytokine levels.

**Fig. 1:**
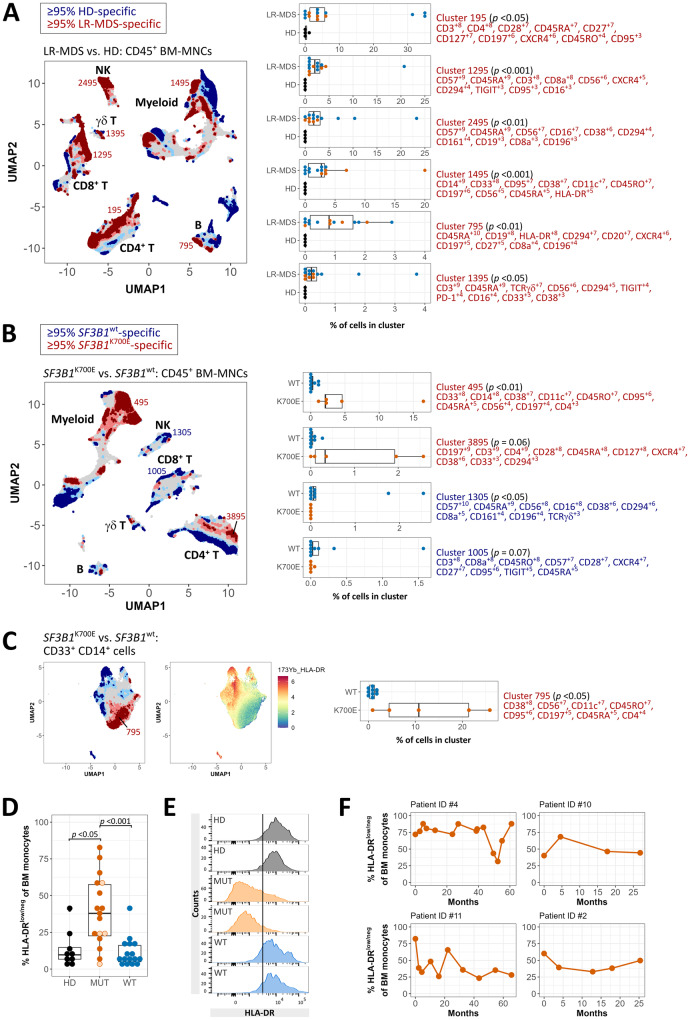
Monocytes with HLA-DR^low/neg^ immunophenotype emerge frequently in the BM of *SF3B1*^mut^ MDS. **A** T-REX plot of regions of significant change on Uniform Manifold Approximation (UMAP) axes for CD45^+^ BM-MNCs stained for CyTOF showing distinct LR-MDS-specific (dark red, ≥95% of cells are contributed by LR-MDS samples) and HD-specific (dark blue, ≥95% of cells are contributed by HD) cell clusters. 14 LR-MDS (mean age = 74 years, 4 women, 10 men) and 4 HD (mean age = 58 years, all men) were included in the analysis. LR-MDS group comprises *SF3B1*^K700E^ (*n* = 5, orange dots; mean age = 75 years, 2 women, 3 men) and *SF3B1*^wt^ (*n* = 9, blue dots; mean age = 74 years, 2 women, 7 men) patients. Top 10 Marker Enrichment Modeling (MEM) labels with enrichment scores are shown for statistically significant LR-MDS-specific clusters (cutoff  >2000 cells) indicated on T-REX plot. **B** T-REX analysis of CD45^+^ BM-MNCs stained for CyTOF showing distinct *SF3B1*^K700E^-specific (dark red) and *SF3B1*^wt^-specific (dark blue) cell clusters. Top 10 MEM labels are shown for statistically significant and trend clusters (cutoff  >1000 cells) indicated on T-REX plot. **A**, **B** Labels on T-REX plot indicate major immune cell subsets (myeloid cells, NK cells, γδ T cells, CD4^+^ and CD8^+^ T cells, B cells). **C** T-REX analysis of CD33^+^ CD14^+^ pre-gated monocytes showing *SF3B1*^K700E^-specific (dark red) and *SF3B1*^wt^-specific (dark blue) clusters. Cluster 795 depicts a distinct HLA-DR^low/neg^ monocyte subset in *SF3B1*^K700E^ LR-MDS (NOTE: this cluster is not related to cluster 795 shown in (A)). HLA-DR expression was projected onto UMAP axes. **A**–**C** Two-sided Mann–Whitney-*U*-test/Wilcoxon rank-sum test was performed for indicated clusters (*p* < 0.05 was considered significant; *p*-values are shown in brackets). Box plots depict median, IQR (lower and upper hinges), and 1.5 times the IQR (lower and upper whiskers extend to values within 1.5 times the IQR from the hinge). **(D)** Percentage of CD33^+^ CD14^+^ BM monocytes with HLA-DR^low/neg^ immunophenotype in HD (median = 9.7, IQR = 8 [*n* = 9, mean age = 69 years, 6 women, 3 men]), *SF3B1*^mut^ (median = 37.9, IQR = 34.9 [*n* = 17; orange dots, K700E; light orange-filled circles, nonK700E including one K666R, one E622D, one H662Y, and one Y623C; mean age = 71 years, 6 women, 11 men]), and *SF3B1*^wt^ (median = 6.3, IQR = 11.2 [*n* = 16, mean age = 66 years, 8 women, 8 men]) MDS assessed by diagnostic FCM of freshly stained BM samples (Kruskal–Wallis test with Dunn’s post-hoc test [Bonferroni adjusted *p*-values]). **E** Representative HLA-DR staining on CD33^+^ CD14^+^ BM monocytes. The black line indicates the set threshold distinguishing low or negative from high HLA-DR expression. **F** Percentage of CD33^+^ CD14^+^ BM monocytes with HLA-DR^low/neg^ immunophenotype in four *SF3B1*^K700E^ MDS patients over time. Patients #4, #10, and #11 harbor an isolated *SF3B1*^K700E^ mutation.

Next, we conducted high-dimensional mass cytometry (CyTOF) on BM-MNCs and analyzed data using the Tracking Responders EXpanding (T-REX) algorithm to identify immunophenotypic differences associated with LR-MDS and *SF3B1*^K700E^ LR-MDS in particular. As expected, LR-MDS (*SF3B1*^mut^ and *SF3B1*^wt^) showed several immunophenotypic changes consistent with an activated immune response (Fig. 1A, Supplementary Fig. S3/S4), in particular specific clusters resembling terminally differentiated effector memory CD8^+^ T cells (T_TE_/TEMRA, cluster 1295), mature CD57^+^ NK cells (cluster 2495), CD27^+^ IgD^−^ memory B cells (cluster 795), and γδ T cells with an exhausted immunophenotype (cluster 1395). LR-MDS exhibited dysregulated T-cell homeostasis, with fewer naïve CD4^+^ and CD8^+^ T cells, and memory phenotype skewing toward CD8^+^ effector memory (T_EM_) and T_TE_ cells (Supplementary Fig. S4). This is consistent with progressive memory differentiation entailing loss of survival, which could contribute to impaired long-term antitumor immunosurveillance.

We then compared *SF3B1*^K700E^ to *SF3B1*^wt^ LR-MDS using the T-REX pipeline, which identified a *SF3B1*^K700E^-specific cluster comprising CD33^+^ CD14^+^ monocytes (cluster 495, *p* < 0.01) (Fig. 1B, Supplementary Fig. S5). Further analysis of CD33^+^ CD14^+^ BM-MNCs showed that a remarkable proportion of the monocytes in *SF3B1*^K700E^ LR-MDS adopt a HLA-DR^low/neg^ phenotype (Fig. 1C). Importantly, retrospective analysis of diagnostic flow cytometry data (Fig. 1D–E) and external validation in two independent cohorts comprising combined 130 MDS (118 LR-MDS) patients (Supplementary Fig. S6) confirmed an increased frequency of HLA-DR^low/neg^ monocytes in *SF3B1*^mut^ (both *SF3B1*^K700E^ and *SF3B1*^nonK700E^) compared to *SF3B1*^wt^ MDS and HD. The external data support our observation that staining cryopreserved BM-MNCs may underestimate the actual frequency of HLA-DR^low/neg^ monocytes. Additionally, we found a strong correlation between HLA-DR^low/neg^ monocyte frequencies in BM and PB (Supplementary Fig. S7). HLA-DR^low/neg^ monocytes in *SF3B1*^mut^ MDS were classical monocytes (CM) based on the lack of CD16 surface expression (Supplementary Fig. S7). Analysis of longitudinal data from four *SF3B1*^K700E^ MDS patients showed a consistently high frequency of HLA-DR^low/neg^ monocytes (Fig. 1F).

To the best of our knowledge, the only other study directly investigating immunophenotypic features in BM of *SF3B1*^mut^ MDS reported lower expression of CD11b, CD36, and CD64 on monocytes [8]. Another study found a higher frequency of thrombomodulin-expressing CM in MDS subtypes with <5% blasts and RS [11]. The association of *SF3B1* mutations with lower monocyte surface HLA-DR expression identified here may be of clinical relevance, for example in view of the predicted poor response of *SF3B1*^mut^ MDS to IST [6, 7]. Overall, the frequency of HLA-DR^low/neg^ monocytes showed no correlation with blood hemoglobin levels, the Revised International Prognostic Scoring System (IPSS-R) risk classifications, or the mutational burden of *SF3B1* and co-mutated *TET2* or *DNMT3A* (Supplementary Fig. S7). HLA-DR^low/neg^ monocyte frequencies were comparable between transfusion-dependent and –independent *SF3B1*^mut^ LR-MDS (Supplementary Fig. S6). However, HLA-DR^low/neg^ monocytes have known immunoregulatory properties via multiple mechanisms, including effector T-cell inhibition, decreased antigen presentation, and defective dendritic cell maturation [12]. A possible scenario is that the early acquisition of *SF3B1* mutations [13] and the presence of inflammation foster the emergence of HLA-DR^low/neg^ monocytes, which then contribute to counteract and balance inflammatory responses in established *SF3B1*^mut^ MDS. In this context, T-REX also identified a cluster of naïve CD4^+^ T cells specific to *SF3B1*^K700E^ LR-MDS with low expression of the co-stimulatory molecule CD27 (cluster 3895, MEM score CD27^+1^) (Fig. 1B, Supplementary Fig. S4/S5). Thus, although disease-related shifts in CD4^+^/CD8^+^ T-cell differentiation were noticeable irrespective of *SF3B1* mutation status, naïve CD4^+^ T cells in *SF3B1*^K700E^ LR-MDS displayed subtle immunophenotypic differences indicative of less recent activation.

**Fig. 2:**
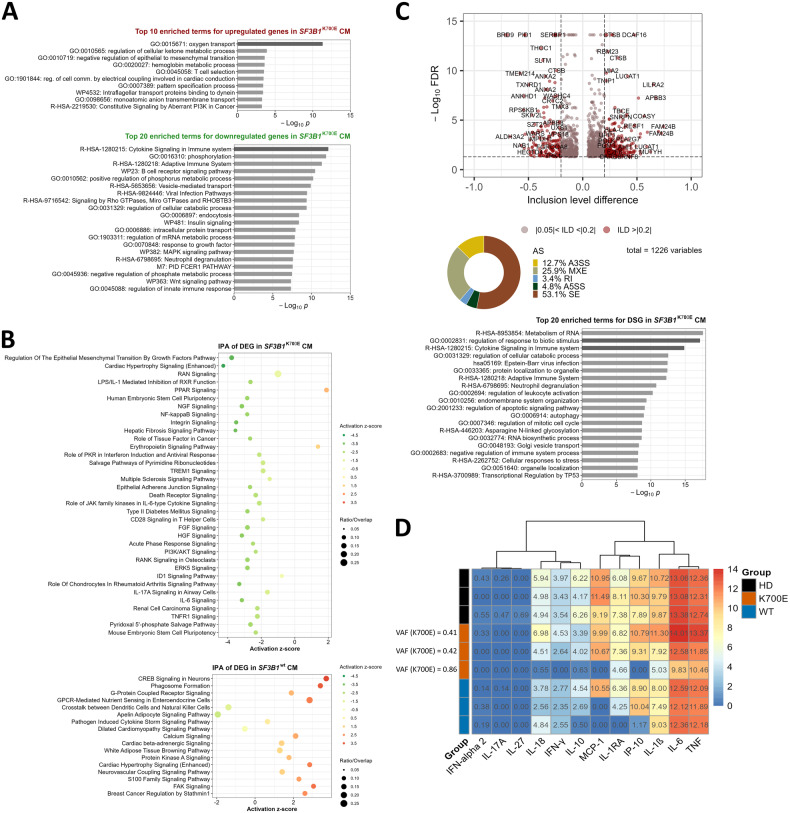
Classical monocytes (CM) from *SF3B1*^K700E^ LR-MDS exhibit dysregulated immune gene expression and splicing. **A** Metascape pathway and process enrichment analysis of up- and downregulated genes in peripheral blood CM from *SF3B1*^K700E^ LR-MDS (*n* = 3) compared to HD (*n* = 3). The top 10 and 20 representative terms are shown for up- and downregulated genes, respectively. **B** IPA core pathway analysis showing the predicted activity (cutoff z-score of >|0.5|) of overrepresented annotations (*p*-value < 0.05 [right-tailed Fisher’s exact test]) based on the list of DEG (PostFC ≥ 2 or ≤0.5, PPDE > 0.95) in *SF3B1*^K700E^ or *SF3B1*^wt^ compared to HD CM (*n* = 3 per group). **C** Alternative splicing (AS) signature in *SF3B1*^K700E^ CM: Volcano plot highlighting differentially spliced genes (DSG) with inclusion level difference (ILD) > |0.2| and pie chart showing distribution of differential splicing event types detectable in *SF3B1*^K700E^ LR-MDS compared to HD CM using rMATS. Shown below is the pathway and process enrichment analysis of DSG using Metascape (top 20 enriched terms across input DSG). **D** Cytokine secretion of LPS-stimulated CM was determined by Luminex analysis. Heatmap depicts Log2-transformed normalized median fluorescence intensity values for the indicated cytokines produced by HD, *SF3B1*^K700E^, or *SF3B1*^wt^ (*n* = 3 per group) LR-MDS classical monocytes following in vitro LPS stimulation. The variant allele frequency (VAF) of *SF3B1*^K700E^ mutation in CM is shown on the left side. AS alternative splicing, A3SS alternative 3’ splice site, A5SS alternative 5’ splice site, DEG differentially expressed genes, DSG differentially spliced genes, MXE mutually exclusive exon, RI retained intron, SE skipped exon.

As CD14^+^ monocytes lose HLA-DR expression, they become functionally deactivated, which can contribute to the transition to a more immunosuppressed state. To investigate whether this is the case for CM from *SF3B1*^K700E^ LR-MDS, we studied their global gene expression profile using RNA-seq. Overall, we found 545 up- and 812 downregulated genes in the clonally involved CM from *SF3B1*^K700E^ LR-MDS compared to HD (Supplementary Table S5). Importantly, these patients harbored an isolated K700E mutation and no confounding cytogenetic aberrations. Upregulated genes were enriched in genes involved in oxygen transport (e.g., *HBB*, *HBA1/*2), probably due to erythrocyte impurities or enhanced phagocytosis of damaged erythrocytes by CM in *SF3B1*^K700E^ LR-MDS. Downregulated genes were significantly enriched in genes related to cytokine signaling, including cytokine receptors (e.g., *IL6R*, *IL10RA*, *IL7R*, *TNFRSF1A*), *TREM1*, signaling kinases (e.g., *MAP3K7*, *MAP3K8*, *PIK3CG*), and NF-κB signaling modulators (e.g., *NFKBIB*, *IKBKG*, *RELA/B*) (Fig. 2A). Ingenuity pathway analysis (IPA) of DEG identified enriched pathways pertaining to inflammatory cytokine signaling (i.e. NF-κB signaling, IL-6 signaling, acute phase response signaling, PI3K/AKT signaling) and inflammatory conditions (i.e. hepatic fibrosis signaling pathway) that could be affected in *SF3B1*^K700E^ CM (Fig. 2B). Expression levels of the NF-κB targets *IL1B* and *TNF* were, however, variable between individual patients (Supplementary Fig. S8). Notably, IPA-based analysis of DEG in CM from *SF3B1*^wt^ LR-MDS patients, of whom 2 out of 3 carried somatic mutations in *TET2*, brought to the fore different inflammatory pathways predicted to be more active compared to HD (Fig. 2B).

In addition, we analyzed alternative splicing in *SF3B1*^K700E^ versus HD CM using rMATS (Supplementary Table S6). Among the more robust differentially spliced genes (DSG) were various genes previously reported as mis-spliced in *SF3B1*^mut^ cells, such as *BRD9*, *COASY*, and *TMEM214* (Fig. 2C, Supplementary Table S6). We could also confirm the previously reported cryptic 3’ splice site for *MAP3K7* predicted to undergo nonsense-mediated RNA decay [14], along with decreased *MAP3K7* transcript levels in *SF3B1*^K700E^ CM (Supplementary Fig. S9, Table S5). We did not observe a clear association of the longer *IRAK4* isoform with *SF3B1*^K700E^ (Supplementary Fig. S9), as has been reported previously [15]. DSG were enriched in genes involved in the regulation of defense response and cytokine signaling, next to mRNA metabolism, apoptotic signaling, and mitotic cell cycle (Fig. 2C, Supplementary Table S7). Importantly, 369 out of the 834 DSG were also differentially spliced in *SF3B1*^K700E^ versus *SF3B1*^wt^ LR-MDS CM (Supplementary Table S8).

Based on the RNA-seq data pointing to dysregulated cytokine signaling in *SF3B1*^K700E^ CM, we then assessed their cytokine secretion following in vitro stimulation with the Toll-like receptor 4 agonist lipopolysaccharide (LPS). We found that CM with a heterozygous mutation in *SF3B1* (VAF ∼ 0.4) responded to LPS stimulation with adequate secretion of pro- (TNF, IL-1β, IL-6, IP-10, MCP-1) and anti-inflammatory cytokines (IL-10, IL-1RA) (Fig. 2D), except for one patient with an extremely high mutation burden (VAF = 0.86) (Fig. 2D). This patient exhibited the highest basal mRNA levels of *TNF* and *IL-6* (Supplementary Fig. S8), which only marginally increased with LPS stimulation. Altogether, at the level of secreted cytokines, we did not observe markedly hyperactivated NF-κB signaling in *SF3B1*^K700E^ CM following LPS exposure, although we can confirm mis-splicing and reduced mRNA expression of *MAP3K7*, previously linked to enhanced NF-kB activity [14]. In interpreting our findings, it is important to acknowledge the small sample size for functional assays as a limitation of our study. Therefore, further research with larger sample sizes will be required to address the functional and stimulation context-dependent deficits resulting from mis-splicing of the identified genes.

Phenotypically, the HLA-DR^low/neg^ CM resemble monocytic myeloid-derived suppressor cells (M-MDSCs), but markers associated with M-MDSC biology were not enriched in *SF3B1*^K700E^ CM (Supplementary Fig. S8). However, HLA-DR^low/neg^ CM from one *SF3B1*^K700E^ LR-MDS patient with co-mutations in *TET2* and *DNMT3A* had a less stimulatory effect on the proliferative capability of autologous CD4^+^ T cells compared to their HLA-DR^high^ counterparts (Supplementary Fig. S10). In light of this, the conversion to HLA-DR^low/neg^ CM may prevent excessive inflammatory reactions in the tissue driven partly by disproportionate T-cell activation. Further studies comparing HLA-DR^low/neg^ and HLA-DR^high^ CM from patients with an isolated *SF3B1*^K700E^ mutation will help to clarify their respective roles in the inflammation process.

### Supplementary information

Supplementary information and data

Supplementary_Table_4

Supplementary_Table_5

Supplementary_Table_6

Supplementary_Table_7

Supplementary_Table_8

## Data Availability

RNA-seq data are publicly available at GEO under accession number GSE236535.
