# Supplementary material for: Mutations in the splicing factor SF3B1 are linked to frequent emergence of HLA-DRlow/neg monocytes in lower-risk myelodysplastic neoplasms
Source: Leukemia. 2024 Apr 17;38(6):1427–31. doi: 10.1038/s41375-024-02249-z (PMC11147767; doi:10.1038/s41375-024-02249-z)
Supplement: Supplementary file 1 — Supplementary information and data [file 41375_2024_2249_MOESM1_ESM.pdf]

SUPPLEMENTAL METHODS

Patient cohort

Overall, BM and PB samples from 166 MDS patients were analyzed for this study. We first analyzed BM/PB samples from 36 newly diagnosed or disease-modifying treatment-naïve (i.e. erythropoietin or supportive care only; deviations from this protocol in four patients are detailed in the legend of Supplemental Figure 1) MDS patients (experimental cohort: 18 *SF3B1*^{mut}, 18 *SF3B1*^{wt}; Supplemental Figure 1 and Supplemental Table 1 summarize somatic mutational data, performed analyses, clinical characteristics) and age-matched healthy donors (HD; BoHemE study [NCT02867085]). We validated findings in two independent cohorts comprising 130 newly diagnosed or disease-modifying treatment-naïve MDS patients (validation cohort 1: 28 *SF3B1*^{mut}, 39 *SF3B1*^{wt}; validation cohort 2: 31 *SF3B1*^{mut}, 32 *SF3B1*^{wt}; Supplemental Figure 6), including HD as reference. MDS and HD biosamples from the experimental cohort were collected with approval from local ethics committees at the University Hospitals Dresden and Leipzig under broad research informed consent with unspecified future use as part of the MDS registry (EK289112008) or BoHemE study (EK393092016, 137/19-lk). The study of biosamples from the validation cohorts was approved by the Independent Ethics Committee of Humanitas Clinical Institute (validation cohort 1: n. 2175/AIRC-IG-2018-Rif. 22053) and the Comitato Etico Area Vasta Centro (validation cohort 2: Em. 2020-298 Rif. CEAVC Studio 6277_oss [già 14.104]).

Supplemental Table 1: Clinical characteristics of MDS experimental cohort

Variable	*SF3B1*^{wt}	*SF3B1*^{mut}	*p*-value
Number of patients	18	18	
Sex			
Female	8	7	
Male	10	11	
Age, years (median)	72.1	72.3	NS
Age range, years	27.5 - 83.5	55.8 - 83.7	
Age IQR, years	61.8 - 75.5	67.7 - 76.8	
RS, % (median)	0	61	<0.001
RS range, %	0 - 75	29 - 89	
RS IQR, %	0 - 7	40 - 71	
BM blasts, % (median)	3	4	NS
BM blasts range, %	0.5 - 16	2 - 13	
BM blasts IQR, %	2.1 - 5.3	2.1 - 4.9	
Hb, g/dL (median)	8.9	8.9	NS
Hb range, g/dL (ref. range 11.92 - 17.24)	7.7 - 13.7	5.8 - 10.3	
Hb IQR, g/dL	8.7 - 10.8	7.9 - 9.6	
WBC, GPt/L (median)	3	4.1	NS (0.06)
WBC range, GPt/L (ref. range 3.8 - 9.8)	0.8 - 6	1.1 - 8.7	
WBC IQR, GPt/L	2 - 4.4	3.1 - 6.5	
ANC, GPt/L (median)	1.7	2.2	NS
ANC range, GPt/L (ref. range 1.8 - 7.55)	0.1 - 4.2	0.4 - 6.4	
ANC IQR, GPt/L	0.7 - 2.8	1.5 - 3.7	
Platelets, GPt/L (median)	185	275	NS
Platelets range, GPt/L (ref. range 150 - 400)	57 - 392	42 - 1471	
Platelets IQR, GPt/L	110 - 267	119 - 358	
ALC, GPt/L (median)	1	1.1	NS
ALC range, GPt/L (ref. range 1.5 - 4)	0.4 - 1.9	0.2 - 2.8	
ALC IQR, GPt/L	0.7 - 1.3	0.7 - 1.4	
AMC, GPt/L (median)	0.25	0.49	<0.01
AMC range, GPt/L (ref. range 0.2 - 1)	0.02 - 0.71	0.17 - 1	
AMC IQR, GPt/L	0.1 - 0.42	0.31 - 0.65	
IPSS-R			
IPSS-R ≤3.5 (number of patients)	13	13	
IPSS-R >3.5 - 4.5 (number of patients)	2	2	
IPSS-R >4.5 (number of patients)	3	3	
CRP, mg/L (median)	4.4	1.7	NS
CRP range, mg/L (ref. range <5)	0.3 - 37.5	0.5 - 18.3	
CRP IQR, mg/L	1.6 - 8.7	0.8 - 3	

Two-sided Mann-Whitney-U-test/Wilcoxon rank-sum test was performed for laboratory variables ($p < 0.05$ was considered significant).

Abbreviations: ALC absolute lymphocyte count, AMC absolute monocyte count, ANC absolute neutrophil count, CRP C-reactive protein, IQR interquartile range, NS not significant, RS ring sideroblasts, WBC white blood cell count

Multiplexed gene expression profiling (NanoString)

Total RNA was isolated with the AllPrep DNA/RNA Kit (QIAGEN, Germantown, MD, USA) from fresh BM mononuclear cells (BM-MNCs) separated by density centrifugation in Ficoll-Paque PLUS (GE Healthcare, Chicago, IL, USA). The expression of 730 immune-related genes was analyzed using the nCounter® PanCancer Immune Profiling Panel (NanoString Technologies, Inc., Seattle, WA, USA). Quality-checked raw data were background-subtracted and normalized using the NanoString nSolver 4.0 software and default settings. Differential expression of genes was inferred by fitting generalized linear models using the R package edgeR (http://bioconductor.org). Genes with false discovery rate (FDR) <0.05 were considered significant. Pathway and process enrichment analysis of differentially expressed genes (DEG) was performed using Metascape[1] (http://metascape.org) as described in Supplemental Methods. The list of DEG was further analyzed by Ingenuity Pathway Analysis (IPA) core analysis (QIAGEN).

CyTOF analysis

Viably frozen BM-MNCs were thawed in 10 mL RPMI medium containing 10% FBS and 1x CTL anti-aggregate wash supplement (CTL-AA-005 CTL, Cellular Technology Limited, USA) and stained with a customized Maxpar Direct Immune Profiling Assay (Standard BioTools Inc., CA, USA; Supplemental Table 2) as described below, employing a CD45-based barcoding approach for multiplex Cytometry by Time-of-Flight (CyTOF).

Following centrifugation (300 x g for 5 min), cells were resuspended in 10 mL CTL wash medium and counted using trypan blue and a hemocytometer. Samples were incubated with 103Rh-Intercalator, washed, and FcR were blocked with 50 µg/sample human IgG blend (KIOVIG, Baxalta Belgium Manufacturing SA/Baxter SA, Belgium). Two differently mass-tagged CD45 antibodies were used to barcode samples prior to their joint surface and intracellular staining. Barcoded samples were stained with APC-conjugated anti-human CD95 for 30 min at 4°C, washed, and transferred into a new tube containing the dried antibody pellet from the Maxpar Direct Immune Profiling Assay. A custom mastermix containing antibodies from the Maxpar Direct T cell Expansion Panel 2 and others as indicated in Supplemental Table 2 was added and cells were incubated for 30 min at RT. After washing, cells were fixed with freshly prepared 1.6% PFA solution for 10 min at RT. Cells were stained intracellularly using the Foxp3/Transcription Factor Staining Buffer Set according to manufacturer's instructions (Thermo Fisher Scientific, USA). Cells were then resuspended in Maxpar Fix & Perm buffer containing Cell-ID™ Intercalator-Ir for nucleated cell discrimination (Standard BioTools Inc., CA, USA) and incubated at 4°C overnight. On the next day, cells were centrifuged and resuspended in 1 mL freezing medium (10% DMSO, 90% FBS) for storage at -80°C until data acquisition. Upon thawing and washing, cells were counted using a Countess® II FL Automated Cell Counter (Thermo Fisher Scientific, USA) before loading for acquisition. All cell washes were performed with Maxpar Cell Staining Buffer.

Samples were acquired on a Helios™ mass cytometer (Standard BioTools Inc., CA, USA). Raw FCS files were normalized using the free CyTOF 6.7 system control software. Data were cleaned, gated on CD45$^+$ cells, and debarcoded using FlowJo v10.8 (FlowJo, Ashland, OR, USA). Due to unspecific staining, the data on Foxp3 were not included. Further gating of key immune cell populations was done using FlowJo. T cell subpopulations were gated based on differential expression of CD45RO, CCR7, CD28, and CD95 according to published gating strategies[2]. Debarcoded CyTOF data (live CD45$^+$ cells or further gated subpopulations as indicated) were concatenated at the group level (LR-MDS [IPSS-R ≤3.5] vs. HD, *SF3B1*^{K700E} vs. *SF3B1*^{wt}) using a customized R-based script, then arcsinh-transformed using a cofactor of 5, and analyzed with the Tracking Responders EXpanding (T-REX) algorithm[3]. T-REX provides a rapid, unsupervised machine learning approach that combines Uniform Manifold Approximation and Projection (UMAP), k-Nearest Neighbour Classification (KNN), and Marker Enrichment Modeling (MEM) to reveal regions of great difference between samples/patients. For our setting, we adapted the publicly available script (https://github.com/cytolab/T-REX) to calculate the percentage of cells within the identified regions for individual patients.

Supplemental Table 2: CyTOF panel

Target	Clone	Metal	Assay/Panel/Source
Anti-human CD45	HI30	89Y	Maxpar Direct Immune Profiling Assay
Live/dead 103Rh-Intercalator	N/A	103Rh	Maxpar Direct Immune Profiling Assay
Anti-human CD196/CCR6	G034E3	141Pr	Maxpar Direct Immune Profiling Assay
Anti-human CD123	6H6	143Nd	Maxpar Direct Immune Profiling Assay
Anti-human CD19	HIB19	144Nd	Maxpar Direct Immune Profiling Assay
Anti-human CD4	RPA-T4	145Nd	Maxpar Direct Immune Profiling Assay
Anti-human CD8a	RPA-T8	146Nd	Maxpar Direct Immune Profiling Assay
Anti-human CD11c	Bu15	147Sm	Maxpar Direct Immune Profiling Assay
Anti-human CD16	3G8	148Nd	Maxpar Direct Immune Profiling Assay
Anti-human CD45RO	UCHL1	149Sm	Maxpar Direct Immune Profiling Assay
Anti-human CD45RA	HI100	150Nd	Maxpar Direct Immune Profiling Assay
Anti-human CD161	HP-3G10	151Eu	Maxpar Direct Immune Profiling Assay
Anti-human CD194/CCR4	L291H4	152Sm	Maxpar Direct Immune Profiling Assay
Anti-human CD25	BC96	153Eu	Maxpar Direct Immune Profiling Assay
Anti-human CD27	O323	154Sm	Maxpar Direct Immune Profiling Assay
Anti-human CD57	HCD57	155Gd	Maxpar Direct Immune Profiling Assay
Anti-human CD183/CXCR3	G025H7	156Gd	Maxpar Direct Immune Profiling Assay
Anti-human CD185/CXCR5	J252D4	158Gd	Maxpar Direct Immune Profiling Assay
Anti-human CD28	CD28.2	160Gd	Maxpar Direct Immune Profiling Assay
Anti-human CD38	HB-7	161Dy	Maxpar Direct Immune Profiling Assay
Anti-human CD56/NCAM	NCAM16.2	163Dy	Maxpar Direct Immune Profiling Assay
Anti-human TCRgd	B1	164Dy	Maxpar Direct Immune Profiling Assay
Anti-human CD294	BM16	166Er	Maxpar Direct Immune Profiling Assay
Anti-human CD197/CCR7	G043H7	167Er	Maxpar Direct Immune Profiling Assay
Anti-human CD14	63D3	168Er	Maxpar Direct Immune Profiling Assay
Anti-human CD3	UCHT1	170Er	Maxpar Direct Immune Profiling Assay
Anti-human CD20	2H7	171Yb	Maxpar Direct Immune Profiling Assay
Anti-human CD66b	G10F5	172Yb	Maxpar Direct Immune Profiling Assay
Anti-human HLA-DR	LN3	173Yb	Maxpar Direct Immune Profiling Assay
Anti-human IgD	IA6-2	174Yb	Maxpar Direct Immune Profiling Assay
Anti-human CD127	A019D5	176Yb	Maxpar Direct Immune Profiling Assay
Anti-human PD-1/CD279	EH12.2H7	165Ho	Maxpar Direct T cell Expansion Panel 2
Anti-APC (Maxpar® Ready)	APC003	106Cd	Biolegend, custom metal labeling
APC anti-CD95	DX2	NA	Biolegend
Anti-human CD33 (Maxpar® Ready)	WM53	111Cd	Biolegend, custom metal labeling
Anti-human OX40/CD134	ACT35	142Nd	Standard BioTools
Anti-human TIM-3/CD366	F38-2E2	159Tb	Maxpar Direct T cell Expansion Panel 2
Anti-human ICOS/CD278	C398.4A	169Tm	Maxpar Direct T cell Expansion Panel 2
Anti-human CD184/CXCR4	12G5	175Lu	Maxpar Direct T cell Expansion Panel 2
Anti-human TIGIT	MBSA43	209Bi	Maxpar Direct T cell Expansion Panel 2
Anti-human CD45	HI30	110Cd	Standard BioTools, used for barcoding
Anti-human CD45	HI30	112Cd	Standard BioTools, used for barcoding

Clinical flow cytometry (FCM) analysis

For validation, we retrospectively analyzed monocytes by expert manual gating of flow cytometric data acquired independently as part of the diagnostic work-up of fresh BM/PB samples (experimental cohort/validation cohort 2) or on cryopreserved BM-MNCs (validation cohort 1).

For the experimental cohort, the monoclonal antibody panels consisted of 8-color-tubes that are part of routine diagnostic flow cytometric analysis of MDS patients. Cell preparation was performed within 24 hours after BM aspiration or PB collection in EDTA tubes. Prior to staining, erythrocytes were removed by bulk lysis for 10 min at RT using BD Pharm Lyse buffer (1:10 dilution with distilled water; BD Biosciences, USA), followed by two washing steps with PBS (Thermo Fisher Scientific, USA). For surface labeling, cells were incubated with monoclonal antibodies (Supplemental Table 3) in the dark (15 min at RT) according to the recommendations of the manufacturer. Subsequently, cells were washed twice and resuspended in 500 µl PBS. Samples were stored at 4°C and acquired within 1 hour on a FACS Canto II cytometer (BD Biosciences) equipped with three lasers (405, 488, and 633 nm). The analysis of FCS files was performed using BD FACSDiva v9.0.1 software. $CD33^{+}$ $CD36^{+}$ monocytes were routinely backgated within CD45/SSC-A to verify that all events fall within the traditional monocyte gate. The same threshold for HLA-$DR^{low/neg}$ $CD14^{+}$ monocytes was set for all samples according to the upper limit of HLA-DR in HLA-DR^{neg} lymphocytes. FlowJo v10.8.1 was used for visualization of data.

Supplemental Table 3: Clinical flow cytometry antibodies

Target	Clone	Source
Anti-human HLA-DR	L243	BD Biosciences
Anti-human CD19	HD37	Agilent Dako
Anti-human CD3	SK7	eBioscience
Anti-human CD16	3G8	Beckman Coulter/Biolegend
Anti-human CD64	10.1	Biolegend
Anti-human CD11b	ICRF44	Biolegend
Anti-human CD14	M5E2/HCD14	BD Biosciences/Biolegend
Anti-human CD36	CB38 (also known as NL07)	BD Biosciences
Anti-human CD33	P67.6	BD Biosciences
Anti-human Lineage Cocktail (CD3, CD14, CD16, CD19, CD20, CD56)	UCHT1, HCD14, 3G8, HIB19, 2H7, HCD56	Biolegend
Anti-human CD45	HI30	BD Biosciences

RNA-seq of classical monocytes and downstream data analysis

Classical monocytes (CM) were isolated from viably frozen PB mononuclear cells (PBMCs) or BM-MNCs. We used a two-step isolation procedure. After depletion of $CD16^{+}$ cells using CD16 MicroBeads, $CD14^{+}$ monocytes were magnetically separated using CD14 MicroBeads over two sequential LS columns (Miltenyi Biotec, Bergisch Gladbach, Germany), yielding routinely >95% $CD14^{+}$ $CD16^{-}$ monocytes based on flow cytometric assessment using a cocktail of monoclonal antibodies (anti-human CD45 [clone 2D1], anti-human CD14 [clone MϕP9], anti-human CD33 [clone P67.6], anti-human CD16 [clone NKP15], anti-human CD34 [clone 8G12], anti-human HLA-DR [clone L243], BD Biosciences). Monocytes (195,000 – 500,000 cells per 96-well in 250 µl RPMI 1640/PenStrep [Life Technologies Corp., NY,

USA]/5% heat-inactivated human serum [Sigma-Aldrich, MO, USA]) were then stimulated with 100 ng/mL LPS (#L3024, Sigma-Aldrich) for 4 hours or left untreated. Following RNA isolation with TRIzol and Direct-zol™ RNA Microprep (Zymo Research, Irvine, CA, USA), mRNA library preparations and sequencing reactions were conducted at GENEWIZ, LLC. (South Plainfield, NJ, USA). The SMART-Seq HT kit (Takara, San Jose, CA, USA) was used for full-length cDNA synthesis and amplification, and Illumina Nextera XT (Illumina, San Diego, CA, USA) was used for sequencing library preparation according to manufacturer's instructions. The samples were sequenced on the Illumina HiSeq 4000 instrument using a 2x150 bp paired-end configuration. Image analysis and base calling were conducted by the HiSeq Control Software. Raw sequence data (.bcl files) generated from Illumina HiSeq was converted into FASTQ files and de-multiplexed using Illumina's bcl2fastq Conversion Software v2.20. Sequencing quality of raw FASTQ files was checked using FastQC 0.11.8 (https://www.bioinformatics.babraham.ac.uk/projects/fastqc/). Adapter trimming and quality filtering of sequence reads were performed using Seqtk and cutadapt. The adapter clipped reads were also aligned to ribosomal RNA sequences using Bowtie 2 (v2.4.1) and the mapped reads were discarded. After that, mapping against the human genome (GRCh38 version 32/Ensembl 98) was performed using STAR aligner v2.7.9a.

SeqPilot software (JSI medical systems GmbH, Ettenheim, Germany) was used for *SF3B1* variant calling using FASTQ files as inputs. RSEM (v1.3.3) was used for quantifying gene and transcript abundances from RNA-seq data. The R package EBSeq[4] (v1.28.0) was used to infer differential gene and isoform expressions. Downstream pathway analysis of DEG was conducted using IPA core pathway and upstream regulator analysis (QIAGEN Ingenuity Systems, CA, USA) by applying the following filters: posterior probability of being differentially expressed (PPDE)>(1-α) (with significance level α set to 0.05) and posterior fold change (PostFC) of ≥2 or ≤0.5. The right-tailed Fisher's exact test was used to estimate the probability that an association between a set of molecules and a biological function or pathway might be due to random chance. The IPA activation z-score was used to infer the activation states ("increased" or "decreased") of implicated biological functions. Replicate multivariate analysis of transcript splicing (rMATS[5] v4.0.2) was used to assess differential alternative splicing events. rMATS detects differential usage of exons by comparing exon-inclusion levels defined with junction reads. Splicing events with FDR <0.05, inclusion level difference of >|0.05|, average read counts ≥10, and average inclusion levels within 0.05 and 0.95 were taken forward (events with missing read count values and events affecting genes on X or Y chromosome were omitted). The EnhancedVolcano and ggvenn R packages were used for data visualization.

In order to confirm and visualize specific alternative splicing events, we re-assessed the RNA-seq data. Reads were processed using a snakemake-based in-house pipeline. Briefly, for mapping we used the splice-aware aligner "STAR" in version 2.7-11b[6] against the human reference genome (GRCh38) and

annotated with the Gencode annotation file, release 42. Transcript-specific expression was then quantified using a reference-guided assembly by applying the assembler StringTie[7] (v2.2.0). Data was visualized using ggsashimi[8] (v1.1.5).

Pathway and process enrichment analysis

Pathway and process enrichment analysis of DEG in BM-MNCs (Supplemental Figure 2) has been carried out using Metascape (http://metascape.org)[1] with the following ontology sources: GO Biological Processes, GO Cellular Components, GO Molecular Functions, KEGG Pathway, and Canonical Pathways. Ontology sources for Metascape pathway and process enrichment analysis of DEG and DSG in CM (Figure 2) included: KEGG Pathway, GO Biological Processes, Reactome Gene Sets, Canonical Pathways, CORUM, WikiPathways, and PANTHER Pathway. Significant enrichment was indicated by a minimum overlap of 3, *p*-value cut-off of 0.01, and minimum enrichment of 1.5. The most significant term within a cluster was chosen to represent the cluster.

Luminex analysis of secreted cytokines

Cytokine levels in supernatants were determined in duplicate using a customized Luminex™ panel for IFN-α2, IFN-γ, IL-1β, IL-1RA, IL-6, IL-10, IL-17A, IL-18, IL-27, CXCL10 (IP-10), MCP-1, and TNF-α (Merck Millipore, MA, USA) on a FLEXMAP 3D™ system (Luminex Corp., Austin, TX, USA) according to the manufacturer´s instructions. The background-subtracted averaged median fluorescence intensity (MFI) values for LPS-stimulated samples were corrected for non-stimulated cytokine secretion through subtraction. We added +1 to all values and plotted log2-transformed LPS-induced MFI values using the R package pheatmap.

T cell assays

Autologous $CD4^+$ $CD25^-$ T cells were isolated from viably frozen PBMCs using the $CD4^+CD25^+$ Regulatory T Cell Isolation Kit (Miltenyi Biotec, Bergisch Gladbach, Germany). Briefly, the flow-through containing the enriched $CD4^+$ $CD25^-$ T cells was used for experiments. $CD4^+$ $CD25^-$ T cells were labeled with the CellTrace™ CFSE Cell Proliferation Kit (Thermo Fisher Scientific, USA) according to manufacturer's instructions. 75,000 CFSE-labeled autologous $CD4^+$ $CD25^-$ T cells were co-cultured with FACS-sorted peripheral blood HLA-$DR^{low/neg}$ or HLA-DR^{high} CM at T cell:CM ratios of 1:1 down to 1:0.13 in 96-well tissue culture-treated plates and stimulated with ImmunoCult™ Human CD3/CD28 T Cell Activator (12,5 µL/mL; STEMCELL Technologies, USA) for 4 days. On day 4, cells were stained with antibodies (BD Biosciences) directed against CD25 (clone 2A3), CD4 (clone RPA-T4), and CD45 (clone 2D1). 7-AAD (BD Biosciences) was added for discrimination of apoptotic and necrotic cells. Stained cells were acquired on a FACS Canto II cytometer (BD Biosciences). The proliferation of viable $CD4^+$ T cells was evaluated analyzing their CFSE fluorescence intensity using the Proliferation Tool in FlowJo v10.8 (FlowJo,

Ashland, OR, USA) and reported as division index (DI). Non-stimulated CFSE-labeled and stimulated non-labeled $CD4^+$ T cells served as controls.

Statistical analysis

Statistical analyses were performed using R (v4.2.3, R Foundation for Statistical Computing, Vienna, Austria) and R Studio (v2023.03.0, Posit Software). A *p*-value of <0.05 was considered statistically significant.

SUPPLEMENTAL TABLES PROVIDED SEPARATELY

Supplemental Table 4: List of DEG between *SF3B1*^{mut} and *SF3B1*^{wt} MDS BM samples

Supplemental Table 5: List of DEG between *SF3B1*^{K700E} LR-MDS and HD classical monocytes

Supplemental Table 6: Differential splicing in *SF3B1*^{K700E} LR-MDS compared to HD classical monocytes

Supplemental Table 7: Metascape pathway and process enrichment analysis of DSG identified in *SF3B1*^{K700E} LR-MDS compared to HD classical monocytes using rMATS

Supplemental Table 8: Differential splicing in *SF3B1*^{K700E} compared to *SF3B1*^{wt} LR-MDS classical monocytes

SUPPLEMENTAL FIGURES

Supplemental Figure 1

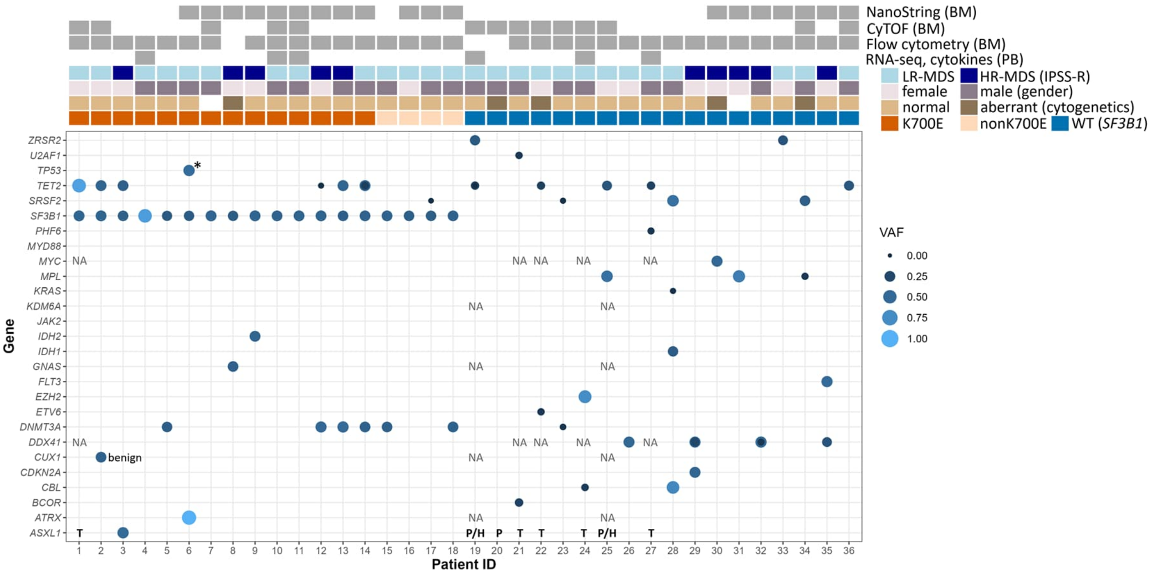

Supplemental Figure 1: Overview of somatic mutational data of MDS patients (experimental cohort) and performed analyses. Summary of somatic mutational data assessed by next generation sequencing showing VAF of gene variants for individual patients. BM samples were screened using the Archer® VariantPlex® Myeloid panel (75 genes) unless otherwise indicated (T, Illumina TrueSight Myeloid Sequencing Panel [54 genes]; P, PB samples used for mutational screening; P/H, PB samples used for mutational screening with a customized Agilent HaloPlex Panel [71 genes]). *SF3B1*^{mut} MDS patients showed a restricted spectrum of co-mutations, with *TET2* and *DNMT3A* being the most frequently mutated co-occurring genes (both 33.3%). Mutations in splicing genes other than *SF3B1* (i.e. *SRSF2*, *U2AF1*, *ZRSR2*) or splicing regulatory genes (i.e. *DDX41*) were detected in 10/18 *SF3B1*^{wt} MDS patients, while 17/18 *SF3B1*^{mut} MDS patients were devoid of mutations in these genes. Annotations above indicate the analyses performed on individual patient samples and clinical characteristics including IPSS-R (LR-MDS, IPSS-R ≤3.5; HR-MDS, IPSS-R >3.5), cytogenetic profile, and *SF3B1* mutation status (K700E, nonK700E, WT). MDS patients were assessed at diagnosis (patients #21, 22, 24, 27) or were disease-modifying treatment-naïve (i.e. EPO, supportive care) with the following deviations: patients #17 and #31 had documented administration of LY2157299 monohydrate in medical history; patient #32 was enrolled in the randomized, placebo-controlled AZA-MDS-003 trial (NCT01566695) before sampling; and patient #36 had documented ATG/CSA 6 years before sampling. *The *TP53* variant detected in patient #6 has uncertain clinical significance and may represent a benign SNP (NM_000546.6(TP53):c.665C>T (p.Pro222Leu))[9].

Supplemental Figure 2

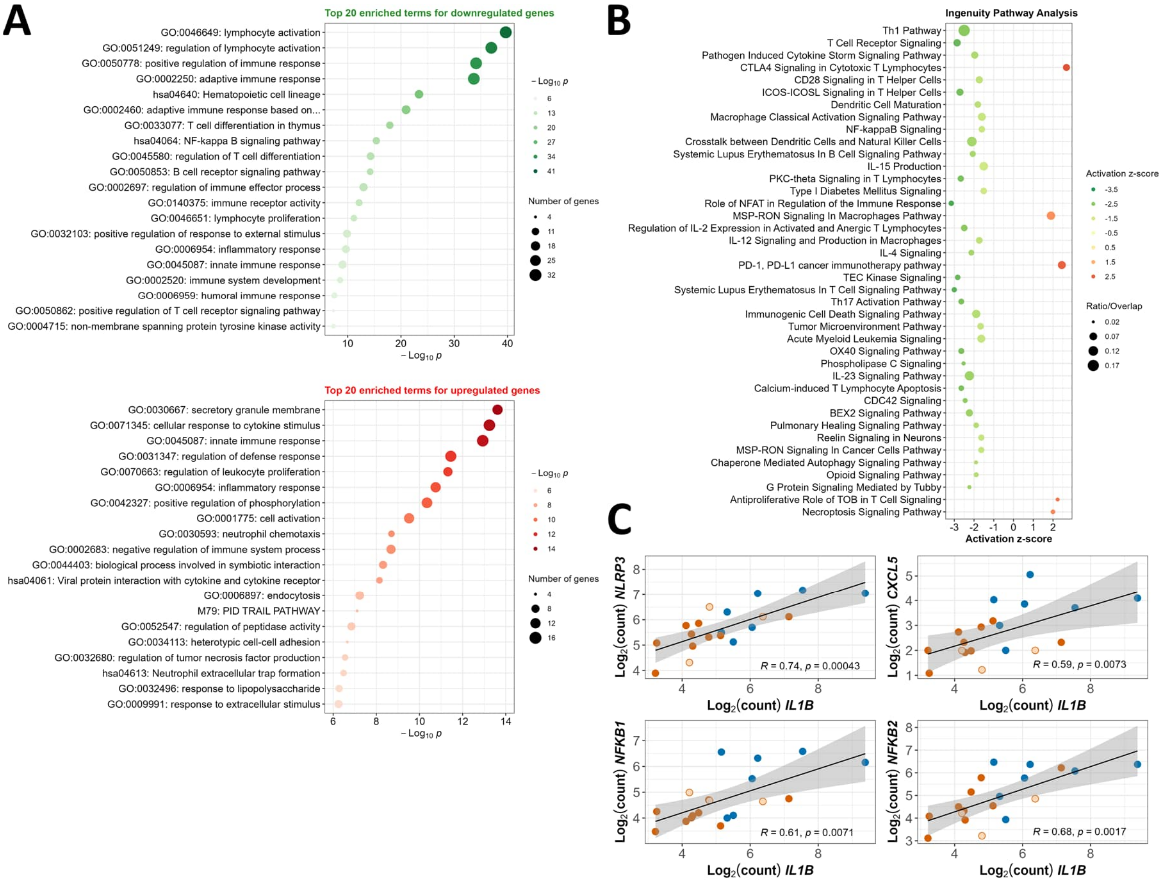

Supplemental Figure 2: Transcriptional immune profiling of BM-MNCs from *SF3B1*^{mut} compared to *SF3B1*^{wt} MDS. (A) Pathway and process enrichment analysis (top 20 representative terms) of down- and upregulated genes in *SF3B1*^{mut} (n=9 *SF3B1*^{K700E} and n=3 *SF3B1*^{nonK700E}, mean age=71 years) compared to *SF3B1*^{wt} MDS (n=7, mean age=62 years) BM samples profiled using the NanoString nCounter® PanCancer Immune Profiling Panel. (B) IPA core pathway analysis showing the predicted activity (cutoff z-score of >|1.5|) of overrepresented annotations (*p*-value <0.05 [right-tailed Fisher's exact test]) based on the list of DEG in *SF3B1*^{mut} compared to *SF3B1*^{wt} BM samples. Z-scores of ≥2 or ≤-2 are considered significant. (C) Correlation of *IL1B* with *NLRP3, CXCL5*, *NFKB1*, and *NFKB2* mRNA expression across *SF3B1*^{mut} (n=12; orange dots, K700E; light orange-filled circles, nonK700E) and *SF3B1*^{wt} BM samples (n=7, blue dots). Scatter plots show Spearman's rank correlation coefficient *R* and *p*-value with linear regression line and 95% confidence interval.

Supplemental Figure 3

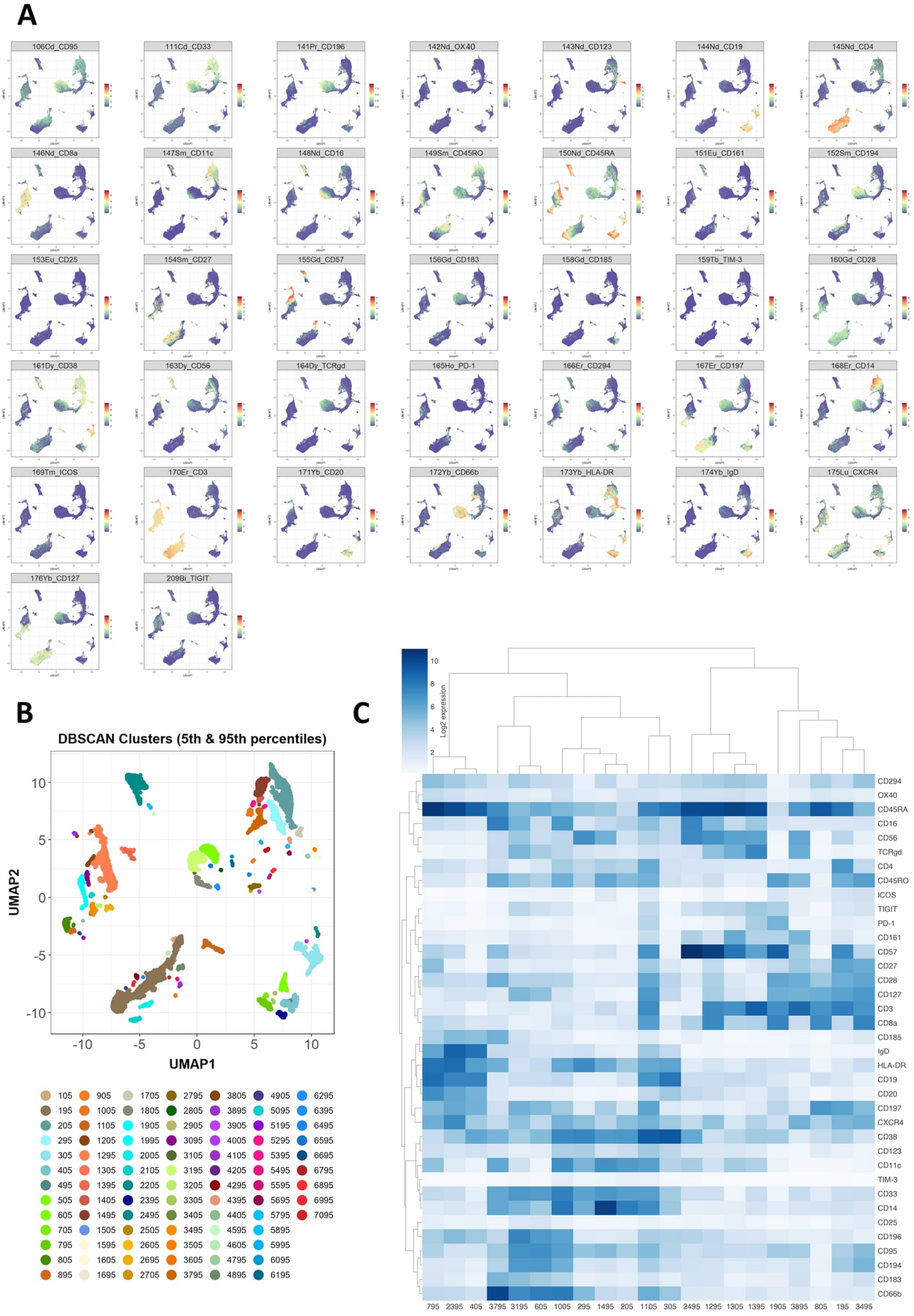

Supplemental Figure 3: T-REX analysis of CyTOF data comparing CD45^{+} BM-MNCs from LR-MDS (n=14) and HD (n=4). (A) Protein marker expression projected onto UMAP axes. (B) Cluster analysis using density-based spatial clustering of applications with noise (DBSCAN) within T-REX workflow. (C) Heatmap depicting the average marker expression (in Log2) across DBSCAN clusters containing >2,000 cells. Clusters were grouped using hierarchical clustering with the complete linkage method and correlation as a distance metric.

Supplemental Figure 4

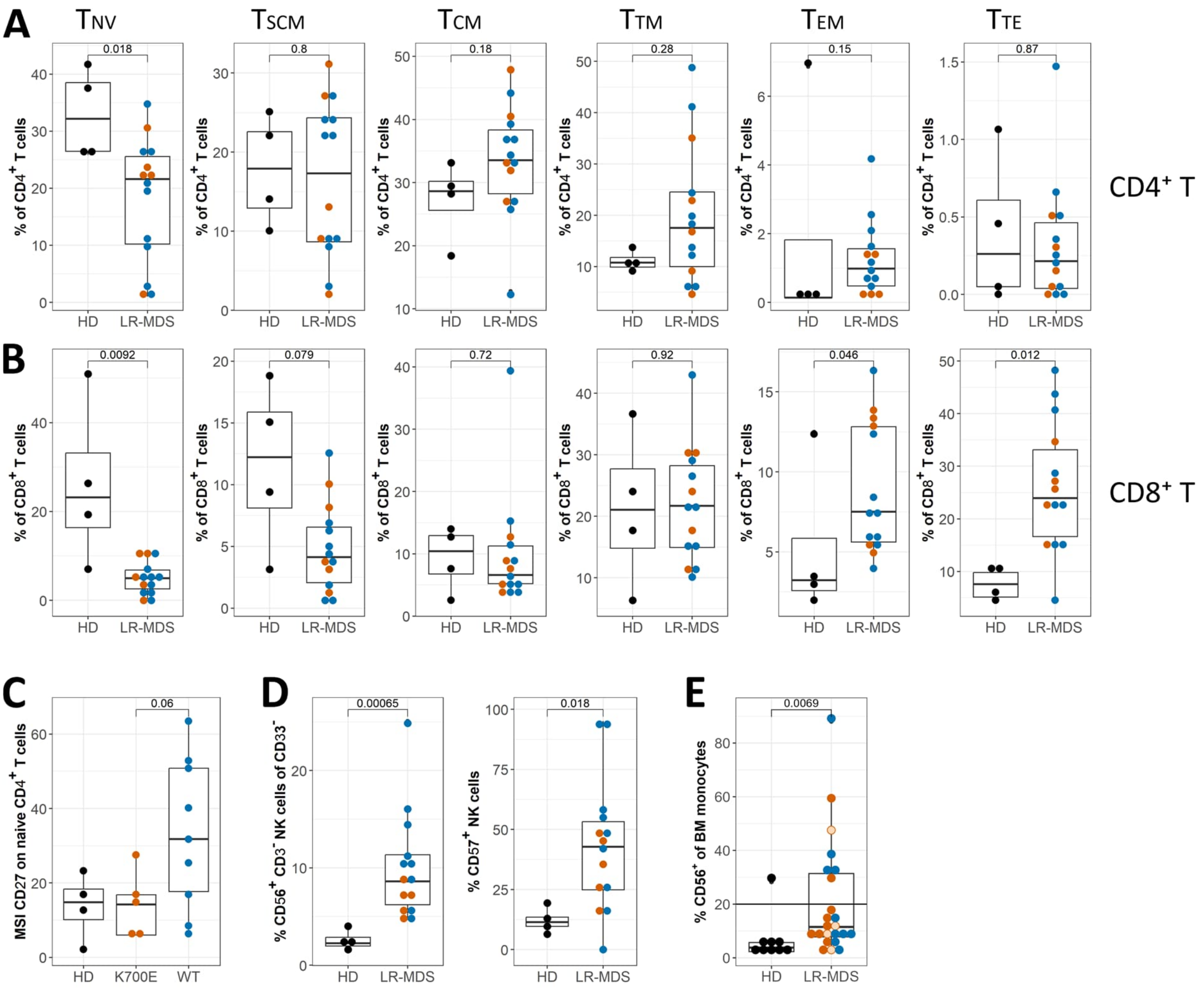

Supplemental Figure 4: Distribution of key immune cell subpopulations in the BM of LR-MDS. (A-D) CyTOF data gated on live CD45^{+} BM-MNCs from HD, *SF3B1*^{K700E} (orange dots), or *SF3B1*^{wt} (blue dots) LR-MDS were further gated for (A, C) CD4^{+} T, (B) CD8^{+} T, and (D) NK cell subsets. (C) Geometric mean signal intensity (MSI) of CD27 expression on naïve CD4^{+} T cells. (D) The frequency of CD56^{+} CD3^{-} NK cells relative to (non-myeloid) CD33^{-} CD45^{+} cells and the proportion of CD57^{+} NK cells. (E) FCM evaluation of aberrant CD56 expression (i.e. ≥20% of monocytes) on freshly stained BM monocytes from HD, *SF3B1*^{K700E} (orange dots, K700E; light orange-filled circles, nonK700E), or *SF3B1*^{wt} (blue dots) LR-MDS. (A-E) Two-sided Mann-Whitney-U-test/Wilcoxon rank-sum test was performed for indicated comparisons (*p*-values are shown above brackets; $p < 0.05$ was considered significant). Box plots depict median, IQR (lower and upper hinges), and 1.5 times the IQR (lower and upper whiskers extend to values within 1.5 times the IQR from the hinge). Abbreviations: T_{CM} central memory T cells, T_{EM} effector memory T cells, T_{NV} naïve T cells, T_{SCM} stem cell-like memory T cells, T_{TE} terminal effector T cells, T_{TM} transitional memory T cells

Supplemental Figure 5

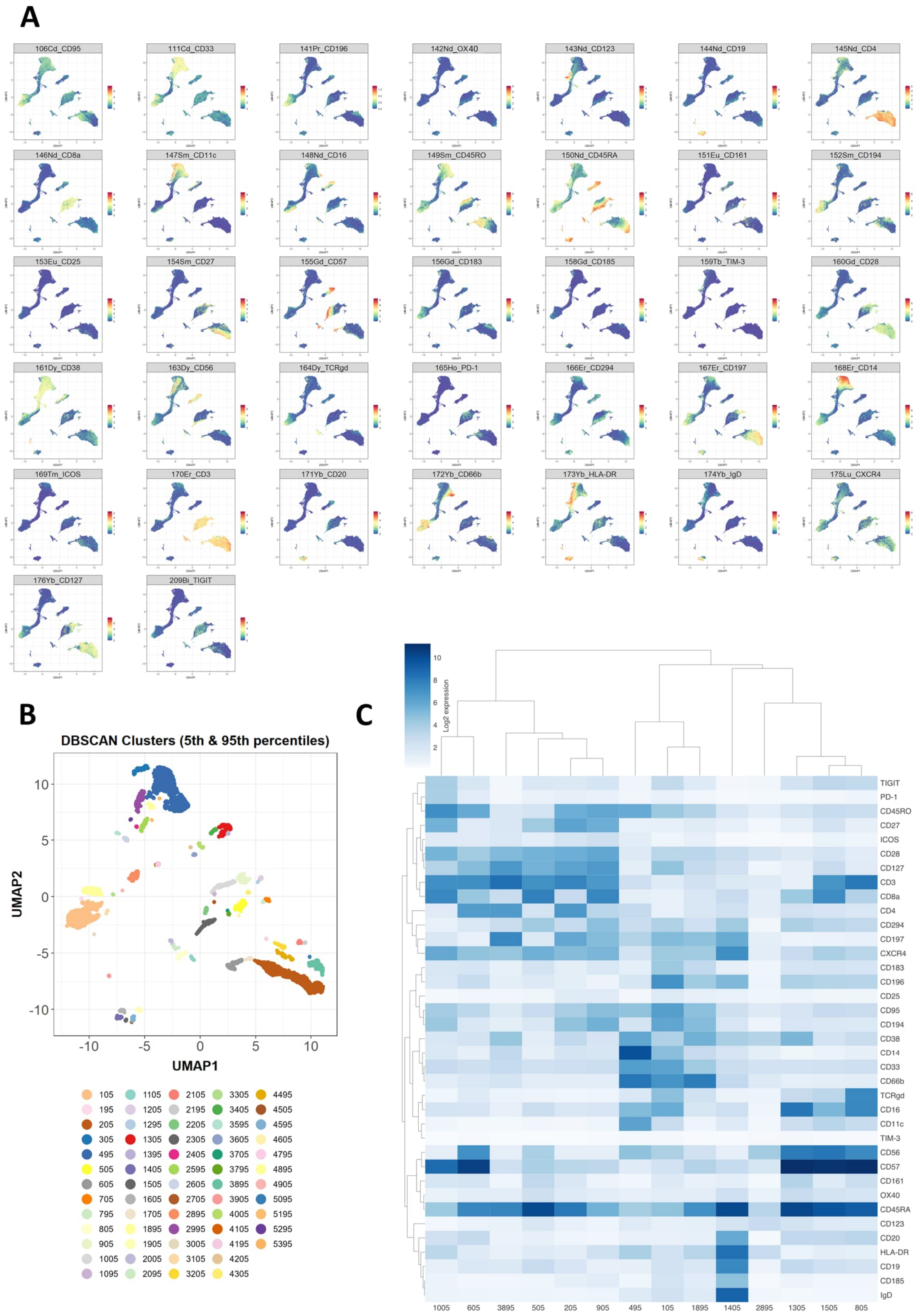

Supplemental Figure 5: T-REX analysis of CyTOF data comparing CD45^{+} BM-MNCs from *SF3B1*^{K700E} (n=5) and *SF3B1*^{wt} (n=9) LR-MDS. (A) Protein marker expression projected onto UMAP axes. (B) Cluster analysis using density-based spatial clustering of applications with noise (DBSCAN) within T-REX workflow. (C) Heatmap depicting the average marker expression (in Log2) across DBSCAN clusters containing >1,000 cells. Clusters were grouped using hierarchical clustering with the complete linkage method and correlation as a distance metric.

Supplemental Figure 6

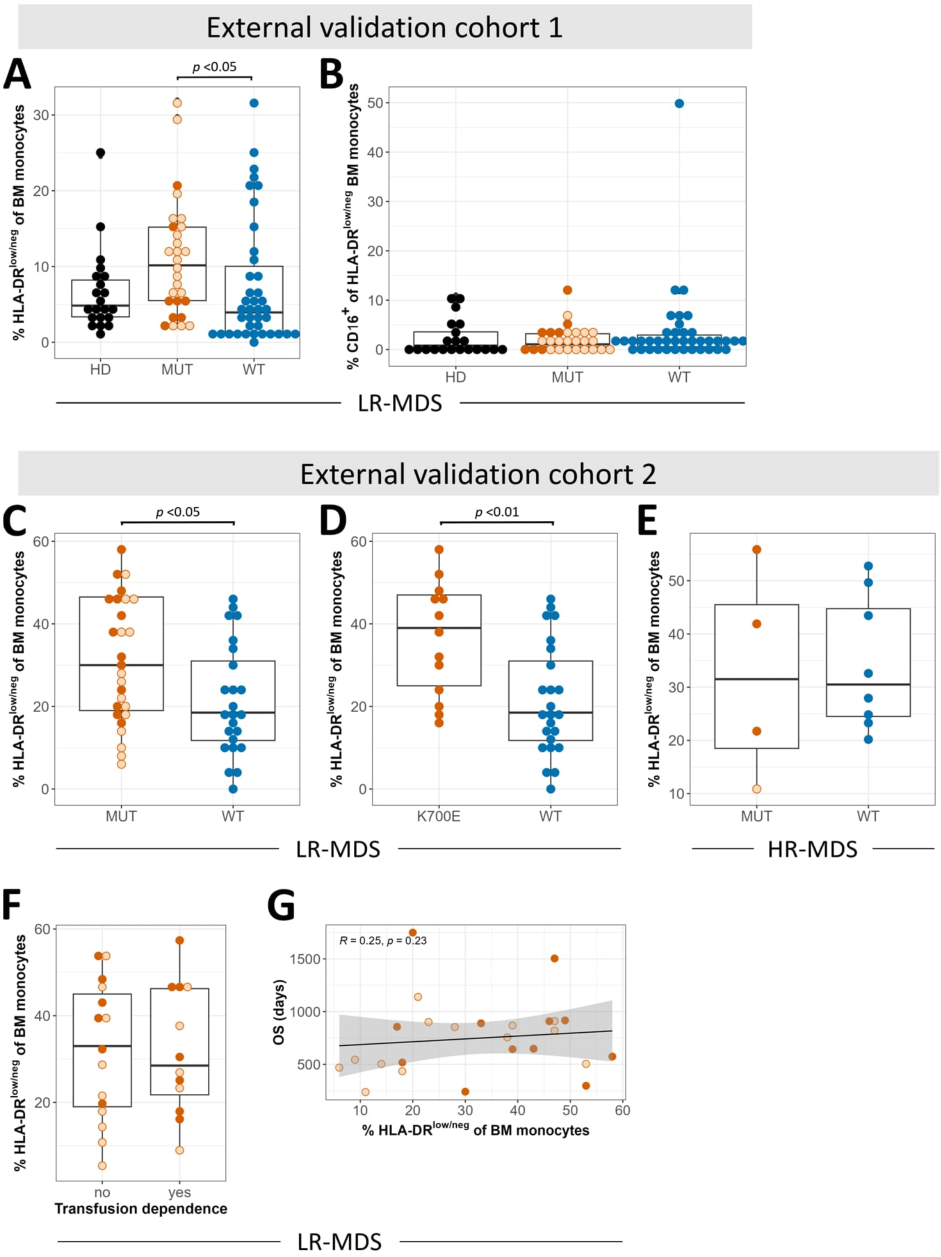

Supplemental Figure 6: External validation confirms increased frequency of HLA-DR$^{low/neg}$ monocytes in *SF3B1*^{mut} compared to *SF3B1*^{wt} LR-MDS. (A-B) Shown are data from external validation cohort 1 comprising 21 HD (mean age=60 years), 28 *SF3B1*^{mut} (orange dots, K700E; light orange-filled circles, nonK700E [two K666, five H662Q, one Y623C, one M784_K785delinsI, one G740E, one E783K, five E622, three R625, one A744P]; mean age=71 years), and 39 *SF3B1*^{wt} (mean age=68 years) disease-modifying treatment-naïve LR-MDS (IPSS-R ≤3.5) assessed by flow cytometry of viably frozen BM-MNCs isolated by density gradient centrifugation in Lympholyte®-H separation medium (Cedarlane). Kruskal-Wallis test with Dunn's post-hoc test (Bonferroni adjusted *p*-values <0.05 were considered significant). Box plots depict median, IQR (lower and upper hinges), and 1.5 times the IQR (lower and upper whiskers extend to values within 1.5 times the IQR from the hinge). (A) Percentage of CD33^{+} CD14^{+} BM monocytes with HLA-DR$^{low/neg}$ immunophenotype. (B) Proportion of HLA-DR$^{low/neg}$ BM monocytes expressing CD16. (C-G) Shown are flow cytometry data of freshly stained BM samples from external validation cohort 2 comprising 31 *SF3B1*^{mut} (orange dots, K700E; light orange-filled circles, nonK700E; mean age=71 years) and 32 *SF3B1*^{wt} (mean age=69 years) patients who were either first-diagnosed or disease-modifying treatment naïve (i.e. EPO in 8 out of 63 patients) LR- and HR-MDS

patients. The gating of HLA-DR$^{low/neg}$ monocytes was performed using Infinicyt flow cytometry software (Cytognos, S.L.) according to the gating strategy applied to the experimental cohort. (C-F) Two-sided Mann-Whitney-U-test/Wilcoxon rank-sum test was performed (p <0.05 was considered significant). Box plots depict median, IQR (lower and upper hinges), and 1.5 times the IQR (lower and upper whiskers extend to values within 1.5 times the IQR from the hinge). (C) Percentage of CD33^{+} CD14^{+} BM monocytes with HLA-DR$^{low/neg}$ immunophenotype in 27 *SF3B1*^{mut} (n=13 *SF3B1*^{K700E}, n=14 *SF3B1*^{nonK700E} [six K666, two T663, three H662Q, two N626D, one E622D]) compared to 24 *SF3B1*^{wt} LR-MDS (IPSS-R ≤3.5). (D) Percentage of CD33^{+} CD14^{+} BM monocytes with HLA-DR$^{low/neg}$ immunophenotype in *SF3B1*^{K700E} compared to *SF3B1*^{wt} LR-MDS. (E) Percentage of CD33^{+} CD14^{+} BM monocytes with HLA-DR$^{low/neg}$ immunophenotype in 4 *SF3B1*^{mut} (n=3 *SF3B1*^{K700E}, n=1 *SF3B1*^{nonK700E}) compared to 8 *SF3B1*^{wt} HR-MDS (IPSS-R >3.5). (F) Percentage of HLA-DR$^{low/neg}$ BM monocytes in red blood cell (RBC) transfusion-dependent (yes, ≥2 units per 8 weeks) and -independent (no, <2 units per 8 weeks) *SF3B1*^{mut} LR-MDS. (G) Correlation of the percentage of HLA-DR$^{low/neg}$ BM monocytes with overall survival (OS) in *SF3B1*^{mut} LR-MDS. Scatter plots show Spearman's rank correlation coefficient *R* and *p*-value with linear regression line and 95% confidence interval.

Supplemental Figure 7

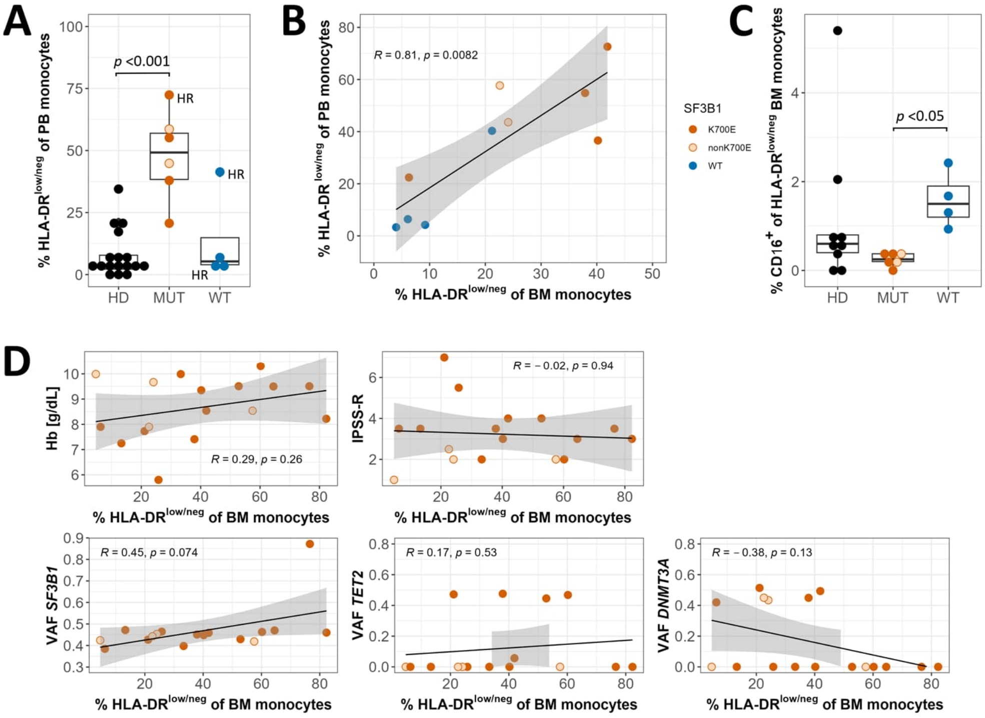

Supplemental Figure 7: Increased proportion of HLA-DR$^{low/neg}$ monocytes in peripheral blood of *SF3B1*^{mut} MDS and further characterization of HLA-DR$^{low/neg}$ BM monocytes. (A) Percentage of PB monocytes with HLA-DR$^{low/neg}$ immunophenotype in HD (n=17, mean age=51 years), *SF3B1*^{mut} (n=6; orange dots, K700E; light orange-filled circles, nonK700E; mean age=74 years), and *SF3B1*^{wt} (n=4, mean age=71 years) MDS assessed by diagnostic flow cytometry of freshly stained PB samples. “HR” labels indicate patients with IPSS-R >3.5. (B) Correlation of frequency of HLA-DR$^{low/neg}$ monocytes in BM and PB. Data show Spearman’s rank correlation coefficient *R* and *p*-value with linear regression line and 95% confidence interval. (C) Proportion of HLA-DR$^{low/neg}$ BM monocytes expressing CD16. (A, C) Kruskal-Wallis test with Dunn’s post-hoc test (Bonferroni adjusted *p*-values). Box plots depict median, IQR (lower and upper hinges), and 1.5 times the IQR (lower and upper whiskers extend to values within 1.5 times the IQR from the hinge). (D) Correlation of the percentage of HLA-DR$^{low/neg}$ BM monocytes with blood hemoglobin (Hb) levels, the Revised International Prognostic Scoring System (IPSS-R) risk classifications, and variant allele frequency (VAF) of *SF3B1* mutation, or confounding *TET2* and *DNMT3A* co-mutations. Scatter plots show Spearman’s rank correlation coefficient *R* and *p*-value with linear regression line and 95% confidence interval.

Supplemental Figure 8

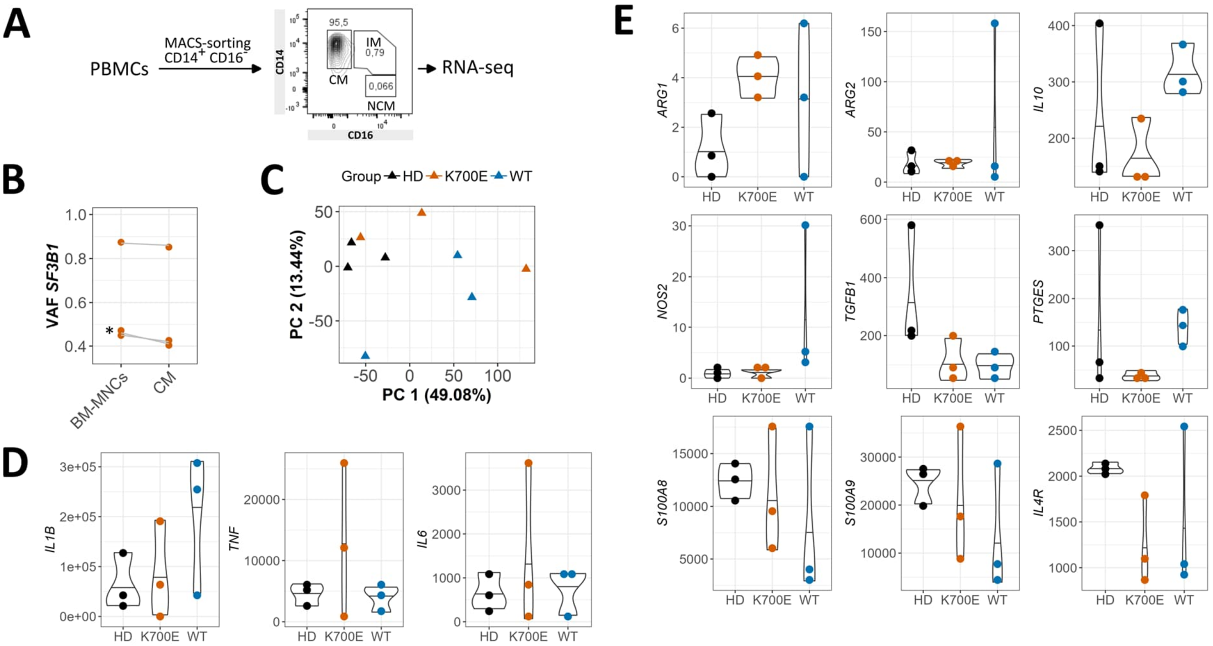

Supplemental Figure 8: RNA-seq of classical monocytes from peripheral blood. (A) Representative flow cytometric evaluation of the purity of MACS-sorted viable CD14⁺ CD16⁻ classical monocytes (CM) and percentages of intermediate (IM) and non-classical (NCM) monocytes. (B) VAF of *SF3B1*^{K700E} mutation in peripheral blood CD14⁺ CD16⁻ CM (RNA-seq data) and paired BM-MNCs (Archer® VariantPlex® Myeloid panel). Asterisk (*) denotes one patient with time lag of 1 year between CM and BM-MNC sampling. Patient with VAF=0.86 has proven somatic *SF3B1*^{K700E} mutation. (C) Principal component analysis of TPM (transcripts per kilobase million) values from RNA-seq of LR-MDS (n=3 *SF3B1*^{K700E} [mean age=70 years, all men], n=3 *SF3B1*^{wt} [mean age=71 years, all men]) and HD (n=3, mean age=66 years, 1 woman, 2 men) classical monocytes. Scatterplot illustrates variation along the first (PC1) and second (PC2) principal component. (D-E) Violin plots of normalized read counts based on RNA-seq data for (D) inflammatory cytokine genes and (E) M-MDSC-associated genes.

Supplemental Figure 9

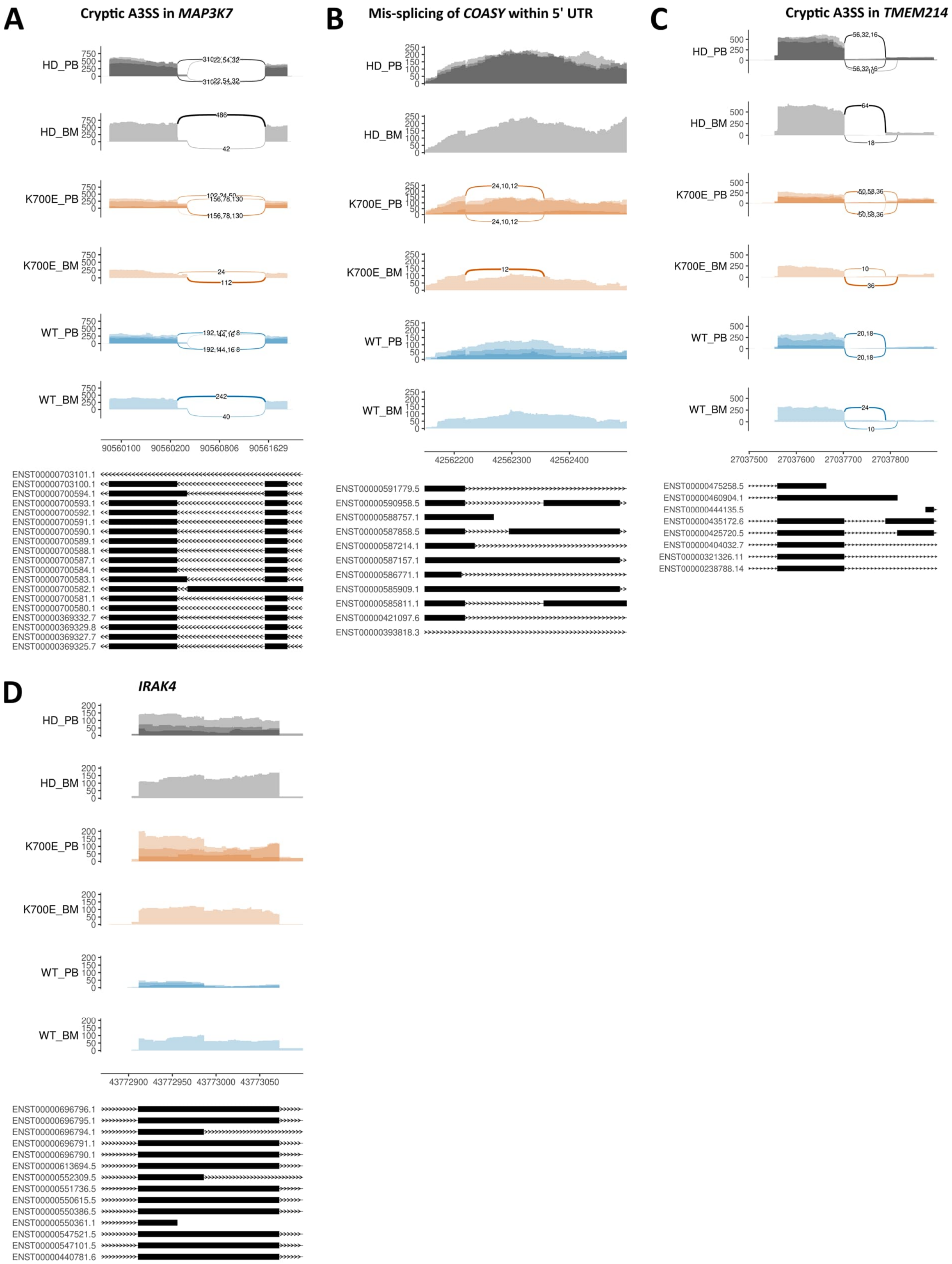

Supplemental Figure 9: Sashimi plots showing read coverage at exon-exon junctions for selected genes in *SF3B1*^{K700E} LR-MDS (orange) compared to HD (grey) and *SF3B1*^{wt} LR-MDS (blue) classical monocytes. Classical monocytes were isolated from PB (n=3 per group) and corresponding BM (n=1 per group) samples. (A) Cryptic 3' splice site (A3SS) in *MAP3K7* as previously published by Lee *et al.*[10], (B) mis-splicing in *COASY* as previously published by Mian *et al.*[11], (C) A3SS in *TMEM214*, and (D) Exon

in *IRAK4* (GRCh38/hg38, chr12: 43772912-43773072) previously described as being preferentially retained in full length in *SF3B1*-mutant compared to HD samples[12]. However, we did not observe this in CM.

Supplemental Figure 10

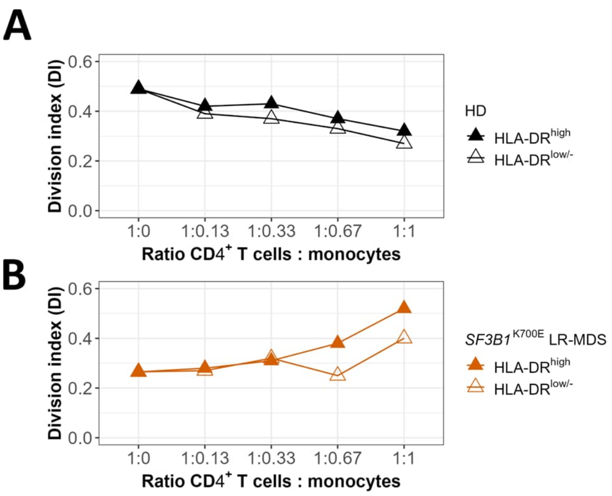

Supplemental Figure 10: Immune regulatory function of HLA-DR$^{low/neg}$ classical monocytes. (A, B) Division index (DI) of CFSE-labeled autologous CD4^{+} T cells stimulated with ImmunoCult^{TM} Human CD3/CD28 T Cell Activator in the absence (1:0) or presence of increasing numbers (1:0.13 up to 1:1) of HLA-DR^{high} (filled triangles) or HLA-DR$^{low/neg}$ (open triangles) classical monocytes isolated from (A) HD or (B) *SF3B1*^{K700E} LR-MDS (patient #14, Supplemental Figure 1).

SUPPLEMENTAL REFERENCES

1 Zhou Y, Zhou B, Pache L, Chang M, Khodabakhshi AH, Tanaseichuk O *et al.* Metascape provides a biologist-oriented resource for the analysis of systems-level datasets. *Nat Commun* 2019; 10: 1523.

2 Mahnke YD, Brodie TM, Sallusto F, Roederer M, Lugli E. The who's who of T-cell differentiation: Human memory T-cell subsets. *Eur J Immunol* 2013; 43: 2797–2809.

3 Barone SM, Paul AGA, Muehling LM, Lannigan JA, Kwok WW, Turner RB *et al.* Unsupervised machine learning reveals key immune cell subsets in COVID-19, rhinovirus infection, and cancer therapy. *Elife* 2021; 10: e64653.

4 Leng N, Dawson JA, Thomson JA, Ruotti V, Rissman AI, Smits BMG *et al.* EBSeq: an empirical Bayes hierarchical model for inference in RNA-seq experiments. *Bioinformatics* 2013; 29: 1035–1043.

5 Shen S, Park JW, Lu ZX, Lin L, Henry MD, Wu YN *et al.* rMATS: Robust and flexible detection of differential alternative splicing from replicate RNA-Seq data. *Proc Natl Acad Sci U S A* 2014; 111: E5593–E5601.

6 Dobin A, Davis CA, Schlesinger F, Drenkow J, Zaleski C, Jha S *et al.* STAR: ultrafast universal RNA-seq aligner. *Bioinformatics* 2013; 29: 15–21.

7 Shumate A, Wong B, Pertea G, Pertea M. Improved transcriptome assembly using a hybrid of long and short reads with StringTie. *PLOS Comput Biol* 2022; 18: e1009730.

8 Garrido-Martín D, Palumbo E, Guigó R, Breschi A. ggsashimi: Sashimi plot revised for browser- and annotation-independent splicing visualization. *PLOS Comput Biol* 2018; 14: e1006360.

9 Soussi T. Benign SNPs in the Coding Region of TP53: Finding the Needles in a Haystack of Pathogenic Variants. *Cancer Res* 2022; 82: 3420–3431.

10 Lee SCW, North K, Kim E, Jang E, Obeng E, Lu SX *et al.* Synthetic Lethal and Convergent Biological Effects of Cancer-Associated Spliceosomal Gene Mutations. *Cancer Cell* 2018; 34: 225-241.e8.

11 Mian SA, Philippe C, Maniati E, Protopapa P, Bergot T, Piganeau M *et al.* Vitamin B5 and succinyl-CoA improve ineffective erythropoiesis in SF3B1-mutated myelodysplasia. *Sci Transl Med* 2023; 15: eabn5135.

12 Choudhary GS, Pellagatti A, Agianian B, Smith MA, Bhagat TD, Gordon-Mitchell S *et al.* Activation of targetable inflammatory immune signaling is seen in myelodysplastic syndromes with SF3B1 mutations. *Elife* 2022; 11: e78136.
